# Spatial models to account for variation in observer effort in bird atlases

**DOI:** 10.1002/ece3.3201

**Published:** 2017-07-18

**Authors:** Andrew M. Wilson, Daniel W. Brauning, Caitlin Carey, Robert S. Mulvihill

**Affiliations:** ^1^ Environmental Studies Department Gettysburg College Gettysburg PA USA; ^2^ Wildlife Management Bureau Pennsylvania Game Commission Harrisburg PA USA; ^3^ Conservation Management Institute Virginia Tech Blacksburg VA USA; ^4^ National Aviary Allegheny Commons West Pittsburgh PA USA

**Keywords:** bird atlas, conditional autoregressive, observer effort, Pennsylvania, spatial model, West Virginia

## Abstract

To assess the importance of variation in observer effort between and within bird atlas projects and demonstrate the use of relatively simple conditional autoregressive (CAR) models for analyzing grid‐based atlas data with varying effort. Pennsylvania and West Virginia, United States of America. We used varying proportions of randomly selected training data to assess whether variations in observer effort can be accounted for using CAR models and whether such models would still be useful for atlases with incomplete data. We then evaluated whether the application of these models influenced our assessment of distribution change between two atlas projects separated by twenty years (Pennsylvania), and tested our modeling methodology on a state bird atlas with incomplete coverage (West Virginia). Conditional Autoregressive models which included observer effort and landscape covariates were able to make robust predictions of species distributions in cases of sparse data coverage. Further, we found that CAR models without landscape covariates performed favorably. These models also account for variation in observer effort between atlas projects and can have a profound effect on the overall assessment of distribution change. Accounting for variation in observer effort in atlas projects is critically important. CAR models provide a useful modeling framework for accounting for variation in observer effort in bird atlas data because they are relatively simple to apply, and quick to run.

## 1 INTRODUCTION

### 1.1 Use of atlases

Grid‐based biological atlases, especially of birds, have become increasingly popular ways of documenting species' status and distributions since the first large‐scale efforts were initiated in the 1960s (Gibbons, Donald, Bauer, Fornasari, & Dawson, 2007). Expanding networks of amateur surveyors have enabled the completion of bird atlas projects covering geographic scales ranging from counties, through states, countries, and even continents (e.g., Hagemeijer & Blair, 1997). Increasingly, bird and other biological atlas data are being utilized to investigate large‐scale environmental issues, notably pertaining to climate change (e.g., Gillings, Balmer, & Fuller, 2015; Huntley, Altwegg, Barnard, Collingham, & Hole, 2012; Matthews, Iverson, Prasad, & Peters, 2011; Thomas & Lennon, 1999; Zuckerberg, Woods, & Porter, 2009) and species conservation (e.g., Araújo, Thuiller, Williams, & Reginster, 2005; Boakes et al., 2010; van der Hoek et al., 2015). Atlas data are especially useful for developing climate envelope models, which can predict future distributions under climate change scenarios, thereby informing large‐scale and long‐term conservations plans (e.g., Beale, Baker, Brewer, & Lennon, 2013; Coetzee, Robertson, Erasmus, Van Rensburg, & Thuiller, 2009; Virkkala & Lehikoinen, 2014).

### 1.2 Variation in effort and false negatives

Because most bird atlas projects rely on citizen scientists to complete the majority of the field surveys, field methods are designed to promote mass participation with the aim of achieving comprehensive spatial coverage (Greenwood, 2007). Inevitably, this leads to trade‐offs between data quality and coverage (Robertson, Cumming, & Erasmus, 2010; Szabo, Butchart, Possingham, & Garnett, 2012), such as the adoption of somewhat flexible field protocols which often do not impose standardization of survey effort. This lack of structure results in spatial variation in observer effort, with the highest effort often expended in areas with habitats or bird communities of most interest to amateur surveyors (e.g., Szabo, Davy, Hooper, & Astheimer, 2007; Tulloch, Mustin, Possingham, Szabo, & Wilson, 2013). Further, accessibility may constrain spatial coverage, such that areas at a greater distance from centers of human populations often receive the lowest effort, especially if there is a lack of accessible roads or paths (McCarthy, Fletcher, Rota, & Hutto, 2012; Syfert, Smith, & Coomes, 2013). Hence, spatial bias can result in significant taxonomic bias, that is, under‐representation of certain species or species groups (Robertson et al., 2010).

When repeat atlases projects are used to assess shifts in species' distributions, changes in survey effort can result in biased measures of changes in range margins (Kujala, Vepsäläinen, Zuckerberg, & Brommer, 2013). It is especially important, therefore, that estimates of changes in distribution between atlas periods include an assessment of changes in survey effort. Many published bird atlases have not accounted for variation in survey effort when producing estimated changes in range size, and indeed, not all bird atlases have adequately collected data to measure survey effort. The application of species distributions models (SDM) is one way of accounting for biases due to variable survey effort. Species distribution models are algorithms that “identify a mathematical or logical function linking species' occurrences and a set of predictors” (Kamino et al., 2012). A multitude of different SDMs have been developed and applied to ecological data (e.g., Aizpurua, Paquet, Brotons, & Titeux, 2015; Comte & Grenouillet, 2013; Elith, Kearney, & Phillips, 2010; Rocchini et al., 2011), but accounting for false negatives is a persistent challenge (Chefaoui & Lobo, 2008; Kéry, 2011; Rocchini et al., 2011). A failure to detect a species in a given area could be because the habitat is not suitable, or it could merely be due to insufficient survey effort or inappropriate survey protocols. Developing survey protocols to maximize the likelihood of detection across multiple species is challenging, and the probability of detecting all species—given their presence—is almost always less than one (Kéry, 2011). Occupancy models (MacKenzie et al., 2006) are increasingly used to account for nonperfect detection in biological atlas data (e.g., Broms, Johnson, Altwegg, & Conquest, 2014). These models are based on capture–recapture theory and hence rely on repeated site visits to model both species occurrence and detectability (MacKenzie et al., 2006). Unfortunately, data captured by bird atlas projects are often highly unstructured and may include opportunistic data, and highly variable sequences of survey effort between blocks, and over multiple years (e.g., Wilson, Brauning, & Mulvihill, 2012). Further, data capture during first generation atlases was often not sophisticated enough to retain a permanent record of the visit history, or even visit years, to each block. Hence, application of occupancy models is not always feasible, and such models can be computationally challenging (Broms et al., 2014; Kéry, Gardner, & Monnerat, 2010), which is a major hurdle if practitioners with limited resources are to apply them to large numbers of species.

### 1.3 Aims

In this study, we use data from the Atlas of Breeding Birds in Pennsylvania, henceforth the “PBBA I” (Brauning, 1992; fieldwork 1983–1989), and the Second Atlas of Breeding Birds in Pennsylvania, henceforth the “PBBA II” (Wilson et al., 2012; fieldwork 2004–2009), to explore the use of a SDM approach to account for variation in observer effort, both within and between repeated atlas projects. We apply the methods to data from the West Virginia Breeding Bird Atlas II project, henceforth WVBBA II (unpublished; fieldwork 2009–2014), to demonstrate that relatively simple conditional autoregressive (CAR) models offer a useful framework for modeling species distributions using atlas data, by incorporating information on broad‐scale landscape features and observer effort. Throughout this study, we use the term “occupancy probability” as shorthand for “occupancy and detection probability.” Our models predict species occurrences based on hypothetical but realistic levels of survey effort per block and hence we do not explicitly model detection probabilities.

Specifically, we test the following hypotheses:

1. Spatial (CAR) models are effective at predicting species' occupancy probabilities for bird atlases with incomplete data
2. Spatial (CAR) models are superior to nonspatial models at predicting species' occupancy probabilities
3. Accounting for variation in observer effort improves model fit

## 2 METHODS

### 2.1 Data

The PBBA I established a survey grid based on U.S. Geological Survey (USGS) 7.5‐min topographic quadrangle maps; dividing each USGS quadrangle into six atlas “blocks,” the bottom‐right of which being designated as a “priority” block. This resulted in 4,937 atlas blocks, including 787 priority blocks, of 3.75' longitude and 2.5' latitude (approximately 5.2 × 4.62 km) across the state of Pennsylvania (Brauning, 1992). Priority blocks were targeted for more thorough coverage than other blocks. Fieldwork was completed by approximately 2,000 volunteers in the years 1983–1989. The PBBA II (2004‐2009) was a repeat effort with very minor changes to field survey protocols. Although the median change in block effort between atlas projects was an increase of 5 hr (mean = 8.68, SD = 33.67), there was not a uniform increase in effort across all blocks. Not only were the changes in effort not evenly distributed across the state (Wilson et al., 2012), but effort was lower than PBBA I in 37% of blocks. It should be noted that details of survey effort in each block (times and duration of individual visits) in PBBA I are longer available, but the database does have a record of total effort hours.

**Figure 1:**
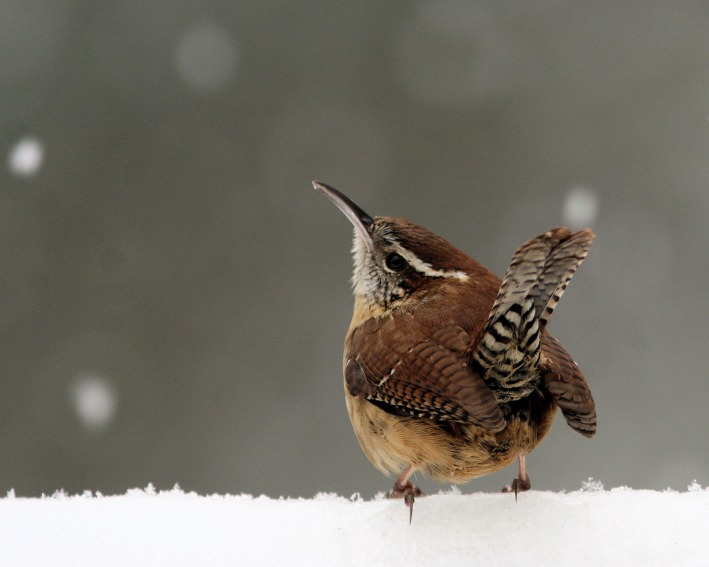
Carolina Wren *Thryothorus ludovicianus* (photo credit: A. Wilson)

**Figure 2:**
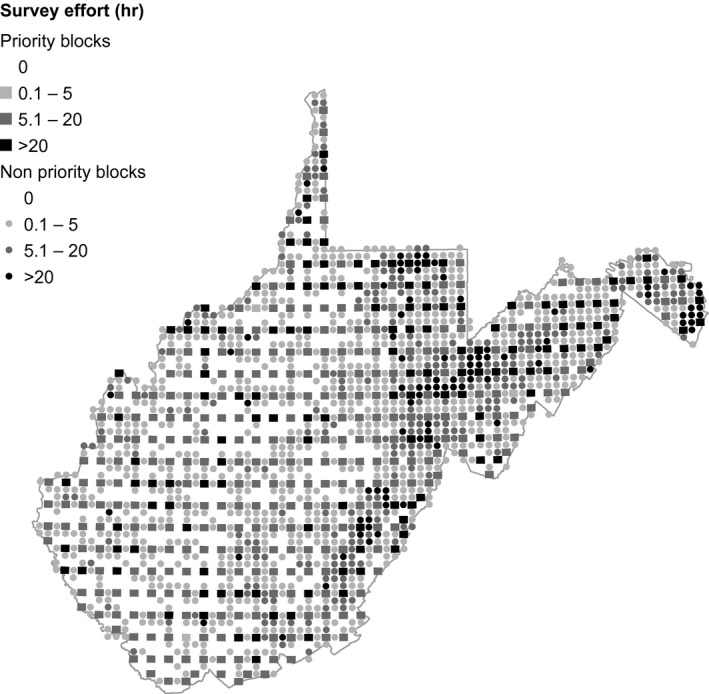
Survey effort in the West Virginia Breeding Bird Atlas II (2009–2014)

Blocks for the WVBBA II (2009–2014), developed for the WVBBA I (1984–1989; Buckelew & Hall, 1994), were delineated in the same way as the PBBA blocks. This resulted in 2,653 atlas blocks across the state of West Virginia, 469 of which were designated as priority blocks. Due to a lower population density, and consequently smaller volunteer pool, block coverage—in 2009–2014—for the WVBBA II was incomplete in comparison with the PBBA II. No bird records were submitted for 580 atlas blocks (21.9%), and observer effort exceeded 1 hr in less than half of blocks (48.2%). However, coverage of the 469 priority blocks in WVBBA II was comprehensive, with a mean of 19.6 hr (SE = 0.65) of survey effort expended per block. Like the Pennsylvania atlas, spatial distributions of observer effort in the WVBBA II were not uniform, consisting of fairly comprehensive coverage in the Allegheny Mountains and lower elevation “panhandle” (eastern half of the state), but very patchy coverage across much of the Allegheny Plateau (western half of the state; Figure 2).

### 2.2 Study species

We tested our hypotheses on Pennsylvania atlas data for six species: Ruffed Grouse (*Bonasa umbellus*), American Kestrel (*Falco sparverius*), Carolina Wren (*Thryothorus ludovicianus*) (Figure 1), Ovenbird (*Seiurus aurocapillus*), Cerulean Warbler (*Dendroica cerulea*), and Henlow's Sparrow (*Ammodramus henslowii*). These species were chosen because they provided a representative sample across abundance and habitat gradients. Among these, six species (Table 1) are localized species (Henslow's Sparrow, found in 4.6% of blocks), species with statewide distributions (Ovenbird, American Kestrel) and range restricted species (Carolina Wren, absent from most of the northern one‐third of the state); forest obligates (Ruffed Grouse, Ovenbird, Cerulean Warbler), a grassland obligate (Henslow's Sparrow), a generalist (Carolina Wren); increasing species (Ovenbird, Carolina Wren) and declining species (Ruffed Grouse, American Kestrel, Cerulean Warbler, Henslow's Sparrow).

**Table 1:** Recorded block detection of the six study species, in both Pennsylvania Atlas periods, and the spatial autocorrelation in block detections (Global Moran's I)

	Block detections (% of all)		Global Moran's I (PBBA II)
	PBBA I	PBBA II	
Ruffed Grouse	2,782 (56.4)	1,870 (37.9)	0.316
American Kestrel	2,938 (60.0)	2,558 (51.8)	0.260
Carolina Wren	2,070 (42.0)	3,487 (70.6)	0.472
Ovenbird	3,674 (74.5)	4,168 (84.4)	0.425
Cerulean Warbler	836 (17.0)	776 (15.7)	0.251
Henslow's Sparrow	364 (7.4)	229 (4.6)	0.246

We used the models developed for the PBBA II to produce predicted probabilities of block occupancy for 136 species (i.e., those that were found in at least 20 blocks) in WVBBA II.

### 2.3 Data analysis

The predicted probability of occurrence by block was modeled using Hierarchical Logistic Regression in WinBUGS for each species (Lunn, Thomas, Best, & Spiegelhalter, 2000). The model took the form:

$$\text{logit}\left(p_{i}\right)\;=\;\alpha \;+\;\left(\sum_{k=1}^{S}\beta_{k}\chi_{ik}\right)\;+\;\gamma_{i}\;+\;\delta_{i}\;+\;\varepsilon$$

where *p*_*i*_ is the predicted probability of occurrence in block *i*, α is the intercept, β_*k*_ is the parameter estimate for *s* landscape covariates χ, γ_*i*_ is a correction factor to account for observer effort (i.e., deviation from a specified “standard”), δ_*i*_ is the spatial effect, and ε is random error.

Landscape covariates were selected by modeling recorded presence/absence using a stepwise (by AIC) logistic model in program R (package MASS; Venables & Ripley, 2002). Between 8 and 12 candidate models were tested for each species. Candidate models were selected based on expert opinion and exploratory analysis of habitat associations (Wilson et al., 2012). There were 26 candidate landscape covariates (Table 2). Land cover data were from Landsat ETM+ derived data (c. 2005; Fry et al., 2011). The extent of reclaimed surface mine grassland was estimated by intersecting areas identified in the Abandoned Mine Inventory data for 2009 (PA DEP, 2009) with grassland and herbaceous land cover types.

**Table 2:** Spatial autocorrelation of landscape covariates among atlas blocks (Global Moran's I). *p *<* *.0001 for all z‐tests

Covariate	Subtype	Mean value (SE)	Global Moran's I	z‐score	Data source
% Forest	All	62.47 (0.37)	0.77	53.47	a
	Deciduous	50.87 (0.39)	0.846	58.82	a
	Mixed	11.08 (0.21)	0.82	57.09	a
	Conifer	0.51 (0.027)	0.66	46.36	a
	Core	34.97 (0.35)	0.752	52.4	a
	Edge	27.51 (0.14)	0.648	45.06	a
	Young forest	0.18 (0.006)	0.548	38.71	a
% Water		1.77 (0.087)	0.304	21.4	a
% Wetland	All	1.29 (0.041)	0.893	62.13	a
	Emergent	1.07 (0.037)	0.892	62.07	a
	Woody	0.22 (0.009)	0.893	62.1	a
% Farmland	All	23.52 (0.29)	0.716	49.77	a
	Grassland	13.66 (0.16)	0.664	46.16	a
	Row crop	9.72 (0.17)	0.736	51.21	a
% Developed	All	10.9 (0.21)	0.757	52.73	a
	Open (e.g., lawn)	6.03 (0.069)	0.689	47.97	a
	Low density	3.04 (0.084)	0.724	50.39	a
	Medium density	1.24 (0.051)	0.665	46.44	a
	High density	0.6 (0.044)	0.673	47.91	a
	Medium + high	1.84 (0.088)	0.695	48.63	a
% Reclaimed strip mines		0.37 (0.021)	0.514	38.74	a,b
Stream density (m/km^2^)		1,072 (4.97)	0.490	34.11	c
River density (m/km^2^)		88.1 (5.92)	0.131	11.34	c
Forested stream density (m/km^2^)		728.8 (4.76)	0.642	44.66	a,c
Mean elevation (m)		377.5 (2.19)	0.923	64.19	d
Elevation range (m)		165.5 (1.37)	0.637	44.34	d

a National Land Cover Data 2006 (Fry et al., 2011).

b Abandoned Mine Land Inventory (PA DEP 2009).

c Networked Streams of Pennsylvania (ERRI 1998).

d National Elevation Dataset, USGS.

Effort effects were modeled after Link and Sauer (2007), using the formula:

$$f\left(\gamma_{i}\right)\;=\;\exp\;\left[D{\left(\gamma_{i}/B\right)}^{c}-1\right]/C$$

where *B* is the standardized number of hours (e.g., mean or median), and *C* and *D* are estimated parameters that determine the shape of the relationship between hours and probability of detection. This formulation enables estimation of effort effects that range from linear *(C *=* *0, *D *=* *1), to diminishing returns (*C *=* *0, 0 < *D *<* *1), and diminishing returns with an asymptote (*C *<* *0, *D *>* *0). This function provides a multiplier; such that if *B *=* *30, the multiplier would be >1 for blocks that receive <30 hr of survey effort—increasing the predicted probability of occurrence in blocks with low effort. In the above example, the multiplier would equal 1 for survey effort of 30 hr.

Spatial effects were included using a Gaussian CAR model, which accounts for spatial autocorrelation in lattice data, such as gridded atlas blocks. Spatially explicit models are “expected to yield better predictions” (Bahn et al., 2006) than nonspatial models. In ecological terms, such models incorporate important spatial information that may relate to unknown effects of bird population dynamics and underlying environmental variation. Spatial autocorrelation was assessed using global Moran's I test statistic using ESRI's Spatial Statistics Tool. A Moran's I value close to zero indicates spatial randomness while a positive value (up to 1) indicates positive spatial autocorrelation. Statistical significance of Moran's I was tested using z‐tests (Z score is based on the Randomization Null Hypothesis computation). The computation of spatial autocorrelation was based on Queen contiguity and Euclidean distance (Anselin, 2005).

Models were implemented using Markov chain Monte Carlo (MCMC) simulation in WinBUGS. Vague normal prior distributions (0, 0.0001) were used to begin the MCMC sampling. Models were fitted with 5,000 iterations following a minimum 1,000 sample burn‐in. Model convergence was checked by examining trace plots for all parameters (Lunn, Jackson, Best, Thomas, & Spiegelhalter, 2012). Most models showed convergence after a few hundred iterations, but models applied to the sparser WVBBA II species data required up to 6,000 iterations to reach convergence.

To cross‐validate our models and test our hypothesis that spatial models are effective at providing predictions for bird atlases with incomplete data, we ran five models for each of the six test species (PBBA II data):

1. all—using data from 100% of atlas blocks
2. 25% random—models trained using 25% of blocks, and tested on the remaining 75%
3. 50% random—models trained using 50% of blocks, and tested on the remaining 50%
4. 75% random—models trained using 75% of blocks, and tested on the remaining 25%
5. Priority blocks—models trained using only priority blocks, which comprise 16.5% of all blocks, and tested on the remaining 83.5%

Model accuracy was evaluated for test data using the area under the receiver operating characteristic (ROC) curve, commonly denoted as area under the curve (AUC). Area under the curve values of 0.5 imply that model accuracy is no better than random, while AUCs of 0.8 or more are considered good, and values of 0.9 or more are considered excellent (Brotons, Herrando, Estrada, Pedrocchi, & Martin, 2008). The AUC for test data was calculated in package pROC of program R (Robin et al., 2011). However, because AUC has received some criticism (e.g., Lobo, Jiménez‐Valverde, & Real, 2008), we included an additional measure of model accuracy: the Point Biserial Correlation coefficient (CORR), which is a special case of Pearson's correlation coefficient that measures the relationship between a continuous and a binary variable, as recommended by Kraemer (2006).

We tested our hypothesis that spatial models are superior to nonspatial models at predicting the presence/absence using four different nested models for each species:

4a (landscape characteristics + spatial effects + effort)
4b landscape characteristics + spatial effects
4c spatial effects + effort
4d landscape characteristics + effort

Models 4a through 4d used the 75% random block data for model training (see model 4). Models were compared using Deviance Information Criterion (DIC).

Our third hypothesis, that incorporating observer effort effects improves model fit, was implicitly tested by comparing models 4a with 4c, and 4b with 4d. We also investigated the relationship between effort and predicted occupancy by running models with varying “standard” amounts of effort (*B*), ranging from 2 hr up to 50 hr, per block; including models with standard effort of 14 (the median block effort in PBBA II) and 21 hr (mean block effort). Effort was modeled as a function of two estimated parameters (after Link & Sauer, 2007), which allowed for a variety of relationships between field survey hours expended and the probability of detecting a species in each block. Hence, the modeled relationship was not necessarily linear; reflecting diminishing returns with increasing effort.

Finally, we demonstrated the extent to which changes in effort substantially affect bird atlas results, by making predictions using a “standard” 40 hr of effort across all blocks in both the PBBA I and PBBA II. A priori, we expected that because overall effort was lower in PBBA I than PBBA II, the effects of modeling presence/absence under a scenario of high effort would increase the number of predicted block occupied in the PBBA I more so than for the PBBA II. Our expectation was therefore that decreases in the number of occupied blocks between the atlases would otherwise have been underestimated, while increases would be overestimated. The models estimating change in the number of occupied blocks included all the data (model 1), and spatial and effort parameters, but did not include landscape covariates (e.g., model 4c) henceforth denoted as model 1c. We chose these reduced models because land cover data were not available for the period of the PBBA I. For further justification of this approach, see the comparison of models 4a through 4d in results. We ran this model for species found in at least 40 blocks during both atlases: 151 species or 88% of the 172 species confirmed to have bred in both PBBA I and PBBA II.

To assess changes in block occupancy between atlas efforts, we used a relative change measures:

$$\begin{array}{cc} & \text{Recorded change in block occupancy}\;\\ & \;=\;\log\left(\frac{\text{recorded block occupancy PBBA II}}{\text{recorded block occupancy PBBA I}}\right) \end{array}$$

and:

$$\begin{array}{cc} & \text{Predicted change in block occupancy}\;\\ & \;=\;\log\left(\frac{\text{predicted block occupancy PBBA II}}{\text{predicted block occupancy PBBA I}}\right) \end{array}$$

## 3 RESULTS

**Figure 3:**
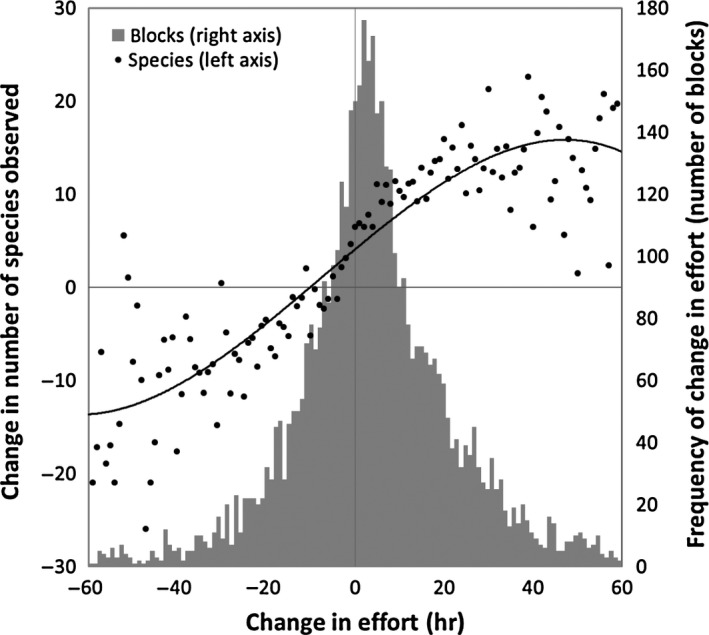
Relationship between change in effort hours and mean change in number of species detected per block, between PBBA I and PBBA II. Trend line fitted is a third‐order polynomial (*R* ^2^ = 0.8). Bars show the frequency of changes in effort hours (changes >60 hr not shown, for clarity)

Data from the two Pennsylvania atlases show that there is a strong relationship between changes in observer effort (hours of effort) and changes in the number of species detected within each block (Figure 3). For blocks where there was a reduction in effort, there was typically a corollary reduction in the number of species observed; while, conversely, the number of species detected usually increased in blocks where effort increased between atlas periods. However, the relationship between changes in effort and changes in observed species richness was not linear, but shows saturation such that increases in effort of more than 40 hr do not continue to accrue these effort effects.

**Figure 4:**
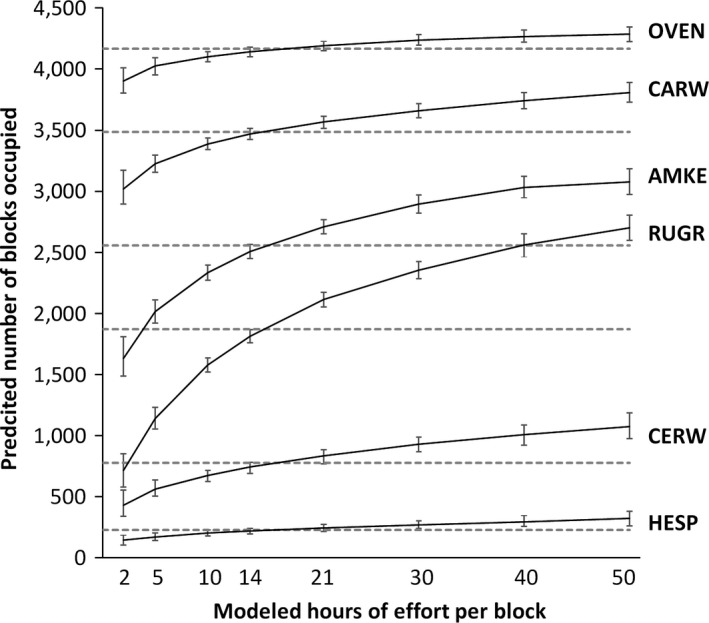
Effects of increasing modeled effort hours on the predicted number of blocks occupied for six study species. Dashed lines represent the actual number of block detection for each species

Underlying the relationship between changes in effort and number of species detected is the fact that the probability of detection for each species is a function of hours of effort expended in each block. Models that corrected PBBA II data for effort show that increased effort hours would significantly increase block detections for all species tested (Figure 4). None of the models reached an asymptote within 50 hr of survey effort, and there were notable differences among species. For Ovenbird, the predicted number of occupied blocks increased slowly with increased survey effort, suggesting that this species was likely to be detected—where present—even with a limited amount of observer effort. In contrast, the predicted number of occupied blocks for Ruffed Grouse markedly increased with increasing effort up to, and beyond, 50 hr. This suggests that Ruffed Grouse were likely substantially underreported in PBBA II, given that mean block effort was 21 hr. Models for the four other study species revealed relationships that fell somewhere between the extremes exhibited by Ruffed Grouse and Ovenbird.

Spatial autocorrelation in PBBA II block occupancy data was highly significant (Z‐test, *p*‐value < .001) for all six study species (Global Moran's I, mean = 0.33; Table 1). Spatial autocorrelation of landscape covariates between atlas blocks was also highly significant (Z‐test, *p*‐value < .0001; Table 2), with Moran's I averaging 0.68 across the 26 covariates tested. Across study species, CAR models were consistently better (higher AUC and CORR) than nonspatial models at predicting the probability of detection in validation (test) blocks (compare models 4a through 4c with 4d, Table 3). However, CAR models that included effort effects but did not include landscape covariates (i.e., model 4c) were either as good as, or better, than the “Full” model (i.e., model 4a) across all six species.

**Table 3:** Comparison of models including landscape covariates, effort effects, and spatial effects, for models using 75% of blocks as training data. Models are compared using Deviance Information Criterion (DIC), the area under the receiving operating characteristic (ROC) curve (AUC), and Point Biserial Correlation Coefficients (CORR) between predictions and recorded block occupancy in the 25% of blocks reserved for validation. Bold indicates best model for each species, based on each of the three metrics

	Model	Model components			Species					
		Landscape Covariates	Spatial Effects	Effort Effects	Ruffed Grouse	American Kestrel	Carolina Wren	Ovenbird	Cerulean Warbler	Henslow's Sparrow
DIC	4a	✓	✓	✓	3023	**3157**	1969	1610	2120	844
	4b	✓	✓		**2930**	3531	1774	1647	2084	963
	4c		✓	✓	2941	3191	**1431**	**958**	**1325**	**531**
	4d	✓		✓	3986	4107	2911	2087	2825	1070
AUC	4a	✓	✓	✓	0.919	0.894	0.961	0.969	0.938	0.953
	4b	✓	✓		0.925	**0.956**	**0.986**	**0.985**	**0.976**	0.939
	4c		✓	✓	**0.927**	0.903	0.964	0.948	0.958	**0.974**
	4d	✓		✓	0.787	0.785	0.876	0.894	0.712	0.940
CORR	4a	✓	✓	✓	0.717	0.687	0.806	0.805	0.703	0.671
	4b	✓	✓		**0.733**	0.702	0.812	0.746	0.763	0.562
	4c		✓	✓	0.730	**0.808**	**0.889**	**0.878**	**0.850**	**0.837**
	4d	✓		✓	0.481	0.493	0.616	0.605	0.325	0.609

**Figure 5:**
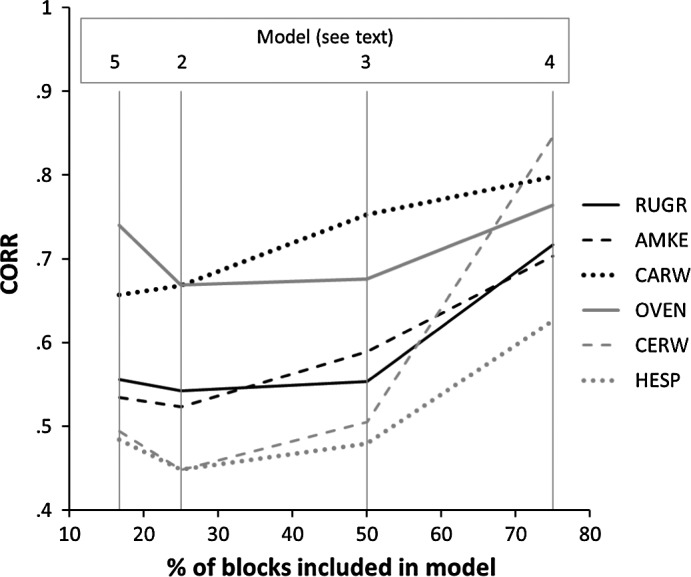
Comparison of predictive performance on test data, as measured by the Point Biserial Correlation coefficient (CORR) of models for six species based on 2nd Pennsylvania Breeding Bird Atlas data (2004–2009)

**Figure 6:**
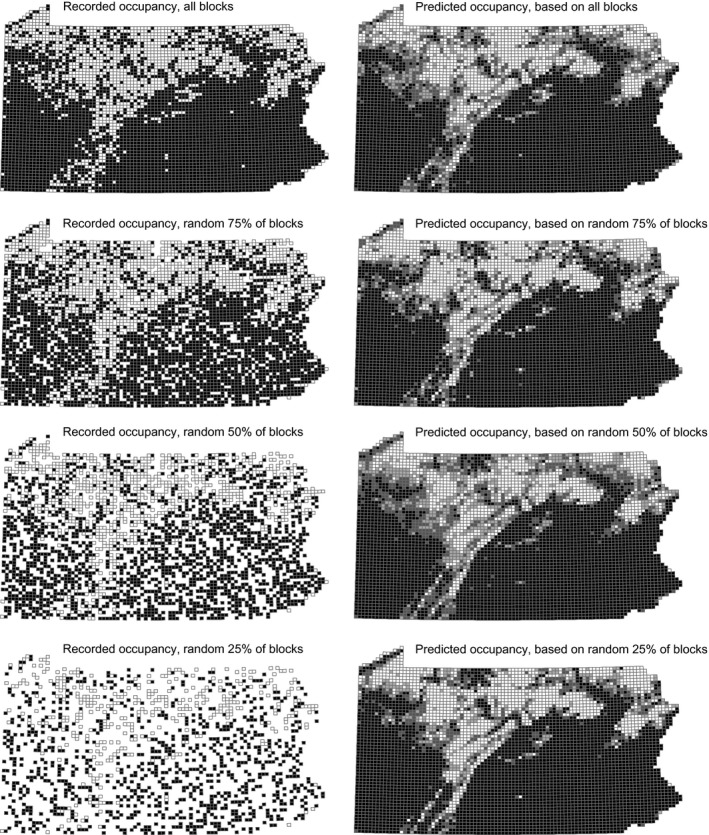
Training data (left) and predicted probabilities of block occupancy for the Carolina Wren in the 2nd Pennsylvania Breeding Bird Atlas. Results of models 1 through 4, top to bottom (see text)

CAR models with landscape and effort effects proved to be good at predicting block occupancy (i.e., high AUC and CORR) for all six test species. Point Biserial Correlation coefficients between observed data and predicted probabilities (training data) were highest for all six species when 75% of block data was used (Figure 5; Table 3). Models based on 75% of block data resulted in the highest AUCs: between 0.898 (American Kestrel) and 0.973 (Carolina Wren). The poorest performing model (American Kestrel, priority blocks) had an AUC of 0.814, still suggesting a “good” model. Maps of predicted block occupancy showed that all models, even those based on only 16.5% and 25% of block data, represented (qualitatively) very reasonable approximations of recorded distributions (see Figures 6 and [Link], [Link], [Link], [Link], [Link]).

Without correcting for effort, the mean relative (recorded) change in block occupancy between the PBBA I and PBBA II among 151 bird species was +6.2% (SE = 1.55), whereas when the overall increase in effort was accounted for (using model 1c), the mean relative predicted change was +3.5% (SE = 1.57). Moreover, by correcting for effort, the predicted change in block occupancy was reduced for 127 of 151 species (either increases were predicted to be lower than recorded, or decreases were predicted to be greater). Without correcting for effort, 109 of 151 species showed an increase in block occupancy, whereas when correcting for effort, only 84 of 151 species showed an increase.

**Figure 7:**
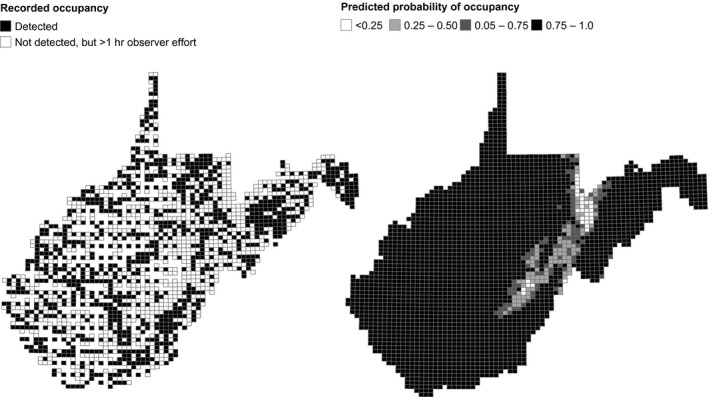
Recorded and predicted probability of block occupancy for the Carolina Wren in the West Virginia Breeding Bird Atlas II

**Figure 8:**
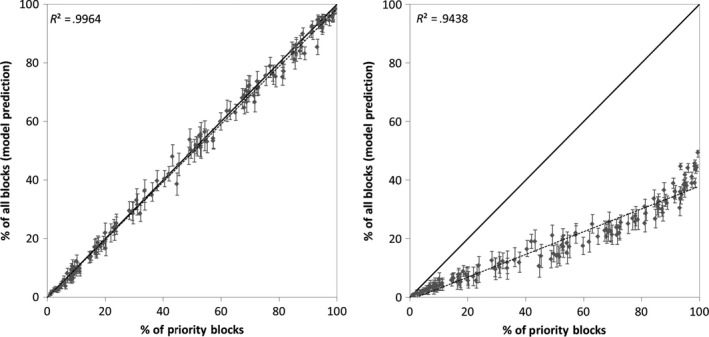
Relationships between block detections in priority blocks and all blocks in the WVBBA II, showing actual data (left), and modeled data (right), for 136 species found in 20 or more atlas blocks. Solid black line is the identity line

Maps of predicted probability of occupancy for the WVBBA II demonstrated that the methods developed for Pennsylvania were especially effective in producing more complete estimates of species distributions for the relatively data sparse WVBBA II (e.g., Figure 7). For example, although the Carolina Wren was documented in just 38.9% of atlas blocks in the WVBBA II, it was detected in 91% of priority blocks, and when modeled, had a predicted block occupancy of 90.4 (95% credible interval 88.7–92.1%). Across all 136 WVBBA II study species, recorded occupancy rates in priority blocks were on average 4.6 times higher than in nonpriority blocks (Figure 8). Further, the relationship between priority and all block (both priority and nonpriority) detection rates was not linear, with under‐detection especially pronounced in moderately widespread species (those found in approx. 25%–75% of priority blocks), as opposed to localized and ubiquitous species (Figure 8). Our models produced predicted block occupancy rates that were very close to those from priority blocks for all species, with the relationship between actual and predicted very close to the identity line (Figure 8).

**Figure 9:**
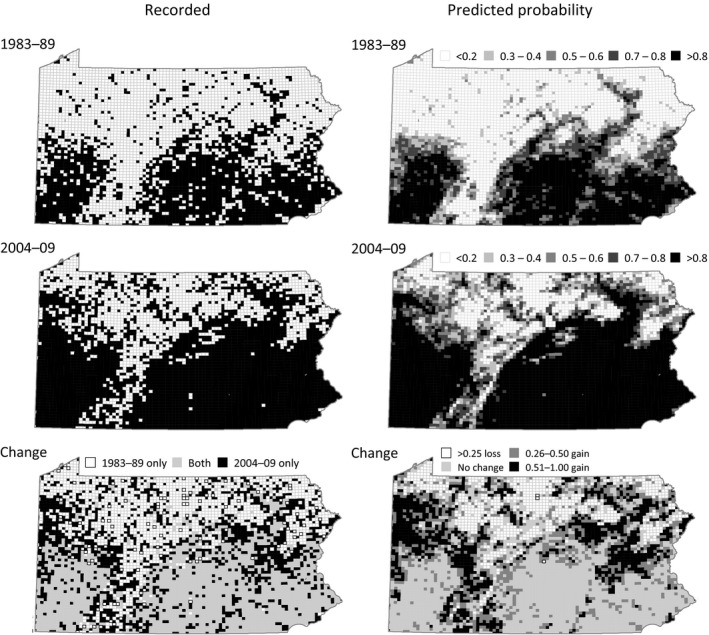
Recorded (left) and predicted (right) distribution of the Carolina Wren in the PABBA I (top) and PABBA II (middle), and change between atlas periods (bottom). Results from model 1 (using all available data, see text)

A map of predicted change in block occupancy of the Carolina Wren in PA between the PABBA I and PABBA II (Figure 9) suggests that most of the isolated instances of apparent loss of block occupancy were likely the result of decreased observer effort in some blocks. The predicted probability of changes in block occupancy provides a clearer map of likely range expansion of this species than the recorded occupancy, even though close to 100% block coverage was achieved in both PABBAI and PABBA II.

## 4 DISCUSSION

Our results suggest that CAR models incorporating coarse landscape and effort effects are successful at predicting species' occupancy probabilities in bird atlas blocks with little to no observer effort. Further, as the landscape covariates added rather little (if any) predictive power for our test species, models incorporating only spatial and effort effects may provide adequate models that circumvent a considerable amount of GIS‐based analysis required to extract landscape covariates, and the subsequent model selection required to identify the best predictors. However, because our model testing was limited to only six species in a relatively homogenous state (all of Pennsylvania is within the Temperate and Broadleaf Mixed Forest Biome; Olsen et al., 2001), we caution against assuming that our findings would apply to all species and regions. The reason that our CAR models had high predictive power, even when landscape covariates were not included, was likely due to the fact that landscape covariates were highly spatially autocorrelated between adjacent atlas blocks; hence, the spatial component of the model accounted for large‐scale patterns in land cover.

The model testing based on various percentages of training data suggests that our CAR models would be applicable to bird atlas projects with incomplete coverage. Even for very sparse data (e.g., 25% training data for Henslow's Sparrow, see Fig. S1.5), our predicted probability of occupancy map provided a good approximation of actual species' distributions. The main failing of our models was an under‐prediction of isolated block occurrences that were outside of the species' core range within the state (e.g., Fig. S1.5). However, it is likely that for many species, isolated block occurrences away from the species' core ranges represented small and temporally erratic populations. Hence, if bird atlas data are to be utilized for conservation planning, correctly demarcating core species' ranges is critical (Rondinini, Wilson, Boitani, Grantham, & Possingham, 2006).

By correcting for survey effort, the number of species assessed that expanded their range (block occupancy) rather than show a range contraction between PBBA I and PBBA II changed sufficiently to put an entirely different complexion on atlas findings. Recorded data suggested that species showing increased block occupancy outnumbered those showing decreased block occupancy by more than two to one (2.59:1), but after correcting for effort, the ratio was much closer to parity (1.25:1). The potential effects of not correcting for survey effort to evaluate range shifts have been documented by others (Kujala et al., 2013). Our analysis supports the need for SDMs that incorporate variation in observer effort (MacKenzie et al., 2006) to correctly measure range shifts.

While our methods show that spatial models can account for variation in observer effort, there are some limitations to our analysis. While the number of effort hours is correlated with the number of species detected in an atlas block, there are several other factors that could influence the probability that any given species is detected, including the efficiency and level of prior experience of observers, the number of individual visits within and between years, the diel distribution of survey effort, and the spatial distribution of effort within a given block. Observer effort may also be influenced by habitat diversity, with more effort required to survey blocks with diverse habitats.

Another potential limitation to our spatial models is the likely presence of anisotropy—that is, directional dependent spatial relationships. The Valley and Ridge Physiographic Province of south‐central Pennsylvania and much of West Virginia has a pronounced southwest to northeast topography, a result of the weathering of belts of rocks from repeated by folding and faulting (Fenneman, 1938). This topography has a direct impact on land use, with farmland and human development dominating the valleys, and forests on the ridges, and hence valleys and ridges have markedly different habitats (Wilson et al., 2012).

Although we included some model selection in our analysis, the multitude of candidate models that can be developed from just a handful of environmental covariates can be daunting, especially when dealing with data from atlas projects that include tens to hundreds of species. Other studies have found that broad land use types, elevation, and (for large extents) latitude and longitude explain a large proportion of the variance in species' distributions (Storch, Konvicka, Benes, Martinkova, & Gaston, 2003). However, our finding that simple spatial models—even those without landscape level covariates—perform well when making predictions based solely on atlas data emphasizes the importance of incorporating spatial autocorrelation into the analysis of atlas data.

While there are many methods available to predict species distributions, including sophisticated methods to account for imperfect detection (e.g., Sadoti, Zuckerberg, Jarzyna, & Porter, 2013), bird atlas projects are often constrained by limited analytical capabilities (i.e., restricted funds to employ data analysts), and a tight deadline to complete analysis for (potentially) 100s of species. In light of those constraints, the relatively simple models used in this study offer a practical alternative. Our models for WVBBA II data typically converged in less than 10 min on a standard desktop computer (Intel Core i7 processor with 3.6 GHz CPU and 16 GB RAM). The rapidity with which these models can be applied would allow for the testing of several competing models, for each species (hence 100s or 1000s of models total) within a relatively short time‐frame.

There has been much discussion about the relative merits of accounting for imperfect detection using occupancy‐detection models (Guillera‐Arroita, 2017). Some studies have shown that occupancy‐detection models perform better for species that are difficult to detect, but that gains are, at best, modest for more easily detected species (Comte & Grenouillet, 2013; Rota, Fletcher, Evans, & Hutto, 2010). Hence, for analysis of atlas data where the main aim is to extrapolate species distributions from incomplete surveys (e.g., WVBBA II), our approach may be sufficient for readily detected species. For less readily detected species, a more sophisticated approach may be necessary, but in those cases, sample sizes (number of block detections) may be prohibitively small, anyway.

### 4.1 Recommendations

Our results suggest that bird atlas data with incomplete block coverage, or uneven effort, can still provide valuable data on species' distributions and distribution change. Relatively simple CAR models provide a usefully modeling framework with which to account for missing data and biases in survey effort. To apply SDM approaches that account for spatial variation in survey effort, it is critical that effort is comprehensively and accurately quantified. Volunteer/surveyor effort hours is now documented by most bird atlas projects, but other ways of measuring effort, such as distance travelled through the sampling unit (Robertson et al., 2010), or species accumulation lists (Moreno & Halffter, 2000). With the increasing use of online data capture for atlas projects (Robertson et al., 2010), the requirement to include a measure of survey effort with each data submission is a simple addition to online data capture forms.

Our analysis of the PABBA II and WVBBA II data revealed that total effort hours may not be sufficient for producing effort‐corrected SDMs for species that are active at specific times of the days, most notably nocturnal species. While the time of day of observations was required, along with overall effort hours in the online data submission portal for PABBA II, we suspect that nocturnal effort hours were under‐reported (Wilson et al., 2012). We therefore suggest that the importance of parsing daytime and nocturnal hours is emphasized in the future atlas efforts, through communication with surveyors and through careful development of recording forms/online portals to document effort hours accordingly. This would also allow for the application of occupancy‐detection models, which may be especially useful for scarce or difficult to detect species (Guillera‐Arroita, 2017).

It is not possible to state a broadly applicable minimum requirement for survey effort and block coverage from our analysis. The minimum requirement would depend to some extent of habitat heterogeneity, species richness, and species' densities. However, the CAR models that we have employed work best when unsurveyed blocks are adjacent to blocks with data—hence, large tracts of unsurveyed blocks should be avoided. We suggest that our methods be applied to different regions, and atlases with a variety of grid sizes and coverage, to assess their general applicability. Finally, we encourage data analysts to report the CPU time required to run SDMs as a matter of course, thereby enabling managers of bird atlases to adequately budget for data analysis following data collection.

## CONFLICT OF INTEREST

None declared.

## References

[ece33201-bib-0001] Aizpurua, O. , Paquet, J.‐Y. , Brotons, L. , & Titeux, N. (2015). Optimising long‐term monitoring projects for species distribution modelling: How atlas data may help. Ecography, 38, 29–40.

[ece33201-bib-0002] Anselin, L. (2005). GeoDa. 0.95i ed. Release Notes. Urbana Champaign, IL: Spatial Analysis Laboratory (SAL), *Department of Agricultural and Consumer Economics*, University of Illinois, USA.

[ece33201-bib-0003] Araújo, M. B. , Thuiller, W. , Williams, P. H. , & Reginster, I. (2005). Downscaling European species atlas distributions to a finer resolution: Implications for conservation planning. Global Ecology and Biogeography, 14, 17–30.

[ece33201-bib-0061] Bahn, V. , O'Connor, R. J. , & Krohn, W. B. (2006). Importance of spatial autocorrelation in modeling bird distributions at a continental scale. Ecography, 29(6), 835–844.

[ece33201-bib-0004] Beale, C. M. , Baker, N. E. , Brewer, M. J. , & Lennon, J. J. (2013). Protected area networks and savannah bird biodiversity in the face of climate change and land degradation. Ecology Letters, 16, 1061–1068. 23782913 10.1111/ele.12139

[ece33201-bib-0005] Boakes, E. H. , McGowan, P. J. K. , Fuller, R. A. , Chang‐qing, D. , Clark, N. E. , O'Connor, K. , & Mace, G. M. (2010). Distorted views of biodiversity: Spatial and temporal bias in species occurrence data. PLoS Biology, 8(6), e1000385 https://doi.org/10.1371/journal.pbio.1000385 20532234 10.1371/journal.pbio.1000385 PMC2879389

[ece33201-bib-0006] Brauning, D. W. (1992). The atlas of breeding birds in Pennsylvania. Pittsburgh, PA: University of Pittsburgh Press.

[ece33201-bib-0007] Broms, K. M. , Johnson, D. S. , Altwegg, R. , & Conquest, L. L. (2014). Spatial occupancy models applied to atlas data show Southern Ground Hornbills strongly depend on protected areas. Ecological Applications, 24, 363–374. 24689147 10.1890/12-2151.1

[ece33201-bib-0008] Brotons, L. , Herrando, S. , Estrada, J. , Pedrocchi, V. , & Martin, J. L. (2008). The Catalan Breeding Bird atlas (CBBA): Methodological aspects and ecological implications. Revista Catalana d'Ornitologia, 24, 118–137.

[ece33201-bib-0009] Buckelew, A. R. , & Hall, G. A. (1994). The West Virginia breeding bird atlas. Pittsburgh, PA: University of Pittsburgh Press.

[ece33201-bib-0010] Chefaoui, R. M. , & Lobo, J. M. (2008). Assessing the effects of pseudo‐absences on predictive model performance. Ecological Modelling, 210, 478–486.

[ece33201-bib-0011] Coetzee, B. W. T. , Robertson, M. P. , Erasmus, B. F. N. , Van Rensburg, B. J. , & Thuiller, W. (2009). Ensemble models predict important bird areas in southern Africa will become less effective for conserving endemic birds under climate change. Global Ecology and Biogeography, 18, 701–710.

[ece33201-bib-0012] Comte, L. , & Grenouillet, G. (2013). Species distribution modelling and imperfect detection: Comparing occupancy versus consensus methods. Diversity and Distributions, 8, 996–1007.

[ece33201-bib-0013] Elith, J. , Kearney, M. , & Phillips, S. (2010). The art of modelling range‐shifting species. Methods in Ecology and Evolution, 1, 330–342.

[ece33201-bib-0014] Environmental Resources Research Institute . (1998). Networked streams of Pennsylvania, vector digital data. University Park, PA: The Pennsylvania State University.

[ece33201-bib-0015] Fenneman, N. M. (1938). Physiography of eastern United States. New York, NY: McGraw‐Hill.

[ece33201-bib-0016] Fry, J. , Xian, G. , Jin, S. , Dewitz, J. , Homer, C. , Yang, L. , … Wickham, J. (2011). Completion of the 2006 national land cover database for the conterminous United States. PE&RS, 77(9), 858–864.

[ece33201-bib-0017] Gibbons, D. W. , Donald, P. F. , Bauer, H.‐G. , Fornasari, L. , & Dawson, I. K. (2007). Mapping avian distributions: The evolution of bird atlases. Bird Study, 54, 324–334.

[ece33201-bib-0018] Gillings, S. , Balmer, D. E. , & Fuller, R. J. (2015). Directionality of recent bird distribution shifts and climate change in Great Britain. Global Change Biology, 21, 2155–2168. 25482202 10.1111/gcb.12823

[ece33201-bib-0019] Greenwood, J. J. D. (2007). Citizens, science and bird conservation. Journal of Ornithology, 148, S77–S124.

[ece33201-bib-0020] Guillera‐Arroita, G. (2017). Modelling of species distributions, range dynamics and communities under imperfect detection: Advances, challenges and opportunities. Ecography, 40(2), 281–295.

[ece33201-bib-0021] Hagemeijer W. J. M., & Blair M. J. (Eds.) (1997). The EBCC atlas of European Breeding Birds: Their distribution and abundance. London: T & A Poyser.

[ece33201-bib-0022] van der Hoek, Y. , Wilson, A. M. , Renfrew, R. , Walsh, J. , Rodewald, P. G. , Baldy, J. , & Manne, L. L. (2015). Regional variability in extinction thresholds for forest birds in the north‐eastern United States: An examination of potential drivers using long‐term breeding bird atlas datasets. Diversity and Distributions, 21(6), 686–697.

[ece33201-bib-0023] Huntley, B. , Altwegg, R. , Barnard, P. , Collingham, Y. C. , & Hole, D. G. (2012). Modelling relationships between species spatial abundance patterns and climate. Global Ecology and Biogeography, 21, 668–681.

[ece33201-bib-0024] Kamino, L. H. Y. , Stehmann, J. R. , Amaral, S. , De Marco Jr, P. , Rangel, T. F. , de Siqueira, J. R. , … Hortal, J. (2012). Challenges and perspectives for species distribution modeling in the neotropics. Biology Letters, 8, 324–326. 22031720 10.1098/rsbl.2011.0942 PMC3367727

[ece33201-bib-0025] Kéry, M. (2011). Towards the modelling of true species distributions. Journal of Biogeography, 38, 617–618.

[ece33201-bib-0026] Kéry, M. , Gardner, B. , & Monnerat, C. (2010). Predicting species distributions from checklist data using site‐occupancy models. Journal of Biogeography, 37, 1851–1862.

[ece33201-bib-0027] Kraemer, H. C. (2006). Correlation coefficients in medical research: From product moment correlation to the odds ratio. Statistical Methods in Medical Research, 15, 525–545. 17260922 10.1177/0962280206070650

[ece33201-bib-0028] Kujala, H. , Vepsäläinen, V. , Zuckerberg, B. , & Brommer, J. E. (2013). Range margin shifts of birds revisited – the role of spatiotemporally varying survey effort. Global Change Biology, 19(2), 420–430. 23504781 10.1111/gcb.12042

[ece33201-bib-0029] Link, W. A. , & Sauer, J. R. (2007). Seasonal components of avian population change: Joint analysis of two large‐scale monitoring programs. Ecology, 88(1), 49–55. 17489453 10.1890/0012-9658(2007)88[49:scoapc]2.0.co;2

[ece33201-bib-0030] Lobo, J. M. , Jiménez‐Valverde, A. , & Real, R. (2008). AUC: A misleading measure of the performance of predictive distribution models. Global Ecology and Biogeography, 17, 145–151.

[ece33201-bib-0031] Lunn, D. , Jackson, C. , Best, N. , Thomas, A. , & Spiegelhalter, D. (2012). The BUGS book ‐ a practical introduction to Bayesian analysis. Boca Raton, FL: CRC Press/Chapman and Hall.

[ece33201-bib-0032] Lunn, D. J. , Thomas, A. , Best, N. , & Spiegelhalter, D. (2000). WinBUGS – a Bayesian modelling framework: Concepts, structure, and extensibility. Statistics and Computing, 10, 325–337.

[ece33201-bib-0033] MacKenzie, D. I. , Nichols, J. D. , Royle, J. A. , Pollock, K. H. , Bailey, L. A. , & Hines, J. E. (2006). Occupancy modeling and estimation. San Diego, CA, USA: Elsevier.

[ece33201-bib-0034] Matthews, S. N. , Iverson, L. R. , Prasad, A. M. , & Peters, M. P. (2011). Changes in potential habitat of 147 North American breeding bird species in response to redistribution of trees and climate following predicted climate change. Ecography, 34(6), 933–945.

[ece33201-bib-0035] McCarthy, K. P. , Fletcher, R. J. Jr , Rota, C. T. , & Hutto, R. L. (2012). Predicting species distributions from samples collected along roadsides. Conservation Biology, 26, 68–77. 22010858 10.1111/j.1523-1739.2011.01754.x

[ece33201-bib-0036] Moreno, C. E. , & Halffter, G. (2000). Assessing the completeness of bat biodiversity inventories using species accumulation curves. Journal of Applied Ecology, 37, 149–158.

[ece33201-bib-0060] Olson, D. M. , Dinerstein, E. , Wikramanayake, E. D. , Burgess, N. D. , Powell, G. V. N. , Underwood, E. C. , … Kassem, K. R. (2001). Terrestrial Ecoregions of the World: A New Map of Life on Earth: A new global map of terrestrial ecoregions provides an innovative tool for conserving biodiversity. BioScience, 51(11), 933–938.

[ece33201-bib-0037] PA DEP . (2009). Abandoned mine land inventory. PA DEP, Bureau of abandoned mine reclamation. Retrieved from http://maps.psiee.psu.edu/preview/map.ashx?layer=459

[ece33201-bib-0038] Robertson, M. P. , Cumming, C. S. , & Erasmus, B. F. N. (2010). Getting the most out of atlas data. Diversity and Distributions, 16, 363–375.

[ece33201-bib-0039] Robin, X. , Turck, N. , Hainard, A. , Tiberti, N. , Lisacek, F. , Sanchez, J.‐C. , & Müller, M. (2011). pROC: An open‐source package for R and S+ to analyze and compare ROC curves. BMC Bioinformatics, 12, 77. 21414208 10.1186/1471-2105-12-77 PMC3068975

[ece33201-bib-0040] Rocchini, D. , Hortal, J. , Lengyel, S. , Lobo, J. M. , Jiménez‐Valverde, A. , Ricotta, C. , … Chiarucci, A. (2011). Uncertainty in species distribution mapping and the need for maps of ignorance. Progress in Physical Geography, 35, 211–226.

[ece33201-bib-0041] Rondinini, C. , Wilson, K. , Boitani, L. , Grantham, H. , & Possingham, H. P. (2006). Tradeoffs of different types of species occurrence data for use on systematic conservation planning. Ecology Letters, 9, 1136–1145. 16972877 10.1111/j.1461-0248.2006.00970.x

[ece33201-bib-0042] Rota, C. T. , Fletcher, R. J. , Evans, J. M. , & Hutto, R. L. (2010). Does accounting for imperfect detection improve species distribution models? Ecography, 34, 659–670.

[ece33201-bib-0043] Sadoti, G. , Zuckerberg, B. , Jarzyna, M. A. , & Porter, W. F. (2013). Applying occupancy estimation and modelling to the analysis of atlas data. Diversity and Distributions, 19(7), 804–814.

[ece33201-bib-0044] Storch, D. , Konvicka, M. , Benes, J. , Martinkova, J. , & Gaston, K. J. (2003). Distribution patterns in butterflies and birds of the Czech Republic: Separating effects of habitat and geographical position. Journal of Biogeography, 30, 1195–1205.

[ece33201-bib-0045] Syfert, M. M. , Smith, M. J. , & Coomes, D. A. (2013). The effects of sampling bias and model complexity on the predictive performance of MaxEnt species distribution models. PLoS ONE, 8(2), e55158. 23457462 10.1371/journal.pone.0055158 PMC3573023

[ece33201-bib-0046] Szabo, J. K. , Butchart, S. H. M. , Possingham, H. P. , & Garnett, S. T. (2012). Adapting global biodiversity indicators to the national scale: A Red List Index for Australian birds. Biological Conservation, 148, 61–68.

[ece33201-bib-0047] Szabo, J. K. , Davy, P. J. , Hooper, M. J. , & Astheimer, L. B. (2007). Predicting spatio‐temporal distribution for eastern Australian birds using Birds Australia's atlas data: Survey method, habitat and seasonal effects. Emu, 107, 89–99.

[ece33201-bib-0048] Thomas, C. D. , & Lennon, J. J. (1999). Birds extend their ranges northwards. Nature, 399, 213.

[ece33201-bib-0049] Tulloch, A. I. T. , Mustin, K. , Possingham, H. P. , Szabo, J. K. , & Wilson, K. A. (2013). To boldly go where no volunteer has gone before: Predicting volunteer activity to prioritize surveys at the landscape scale. Diversity and Distributions, 19, 465–480.

[ece33201-bib-0050] Venables, W. N. , & Ripley, B. D. (2002). Modern applied statistics with S, 4th ed New York, NY: Springer.

[ece33201-bib-0051] Virkkala, R. , & Lehikoinen, A. (2014). Patterns of climate‐induced density shifts of species: Poleward shifts faster in northern boreal birds than in southern birds. Global Change Biology, 20, 2995–3003. 24729475 10.1111/gcb.12573

[ece33201-bib-0052] Wilson, A. M. , Brauning, D. W. , & Mulvihill, R. S. (2012). Second atlas of breeding bird in Pennsylvania. University Park, PA: Pennsylvania University Press.

[ece33201-bib-0053] Zuckerberg, B. , Woods, A. M. , & Porter, W. F. (2009). Poleward shifts in breeding bird distributions in New York State. Global Change Biology, 15, 1866–1883.

