# Supplementary material for: Spatial models to account for variation in observer effort in bird atlases
Source: Ecol Evol. 2017 Jul 18;7(16):6582–94. doi: 10.1002/ece3.3201 (PMC5574789; doi:10.1002/ece3.3201)
Supplement: Supplementary file 3 [file ECE3-7-6582-s003.pdf]

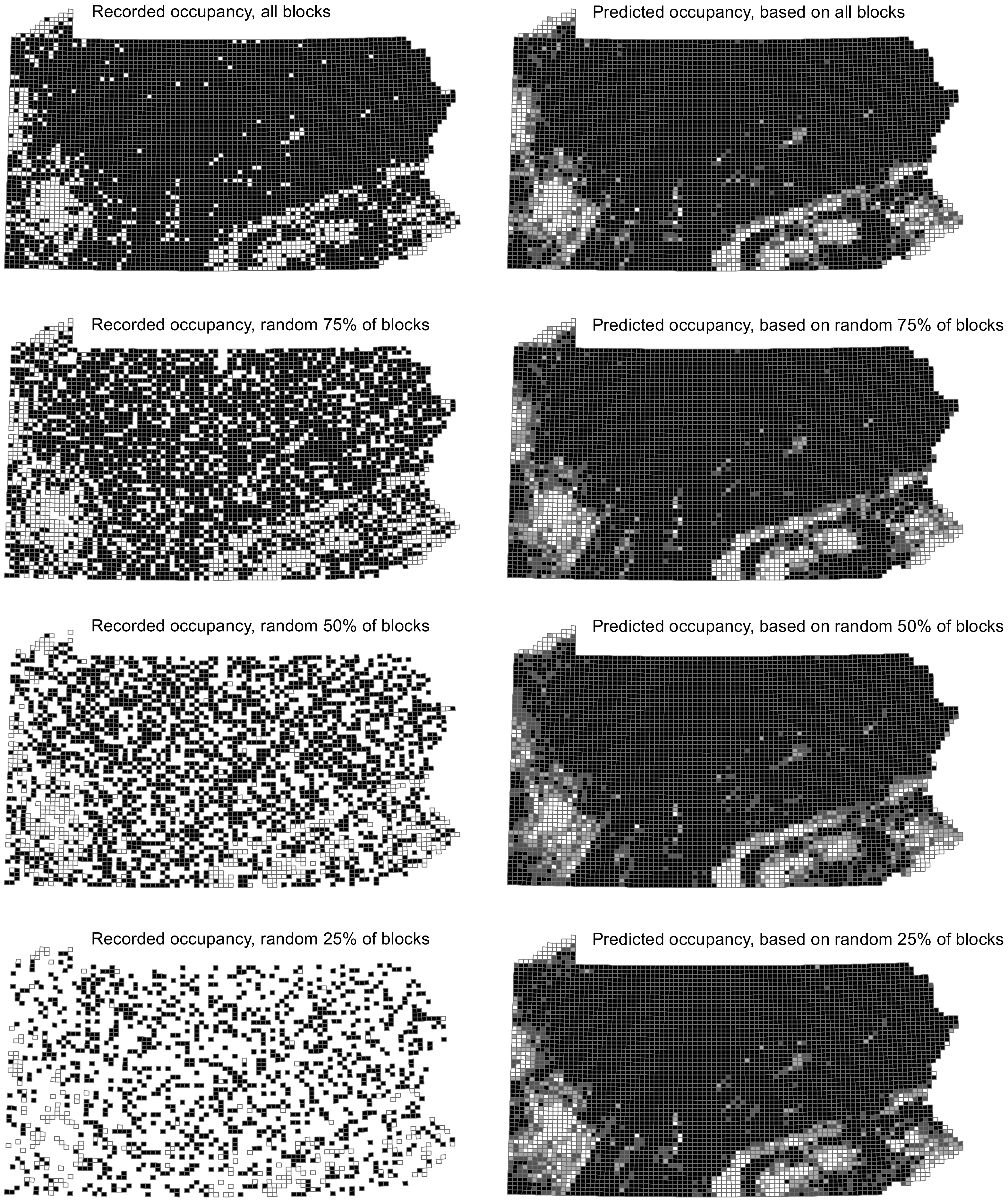
Recorded occupancy, all blocks
Predicted occupancy, based on all blocks
Recorded occupancy, random 75% of blocks
Predicted occupancy, based on random 75% of blocks
Recorded occupancy, random 50% of blocks
Predicted occupancy, based on random 50% of blocks
Recorded occupancy, random 25% of blocks
Predicted occupancy, based on random 25% of blocks
